# Inflammatory myofibroblastic tumour of the common bile duct: a case report and literature review

**DOI:** 10.1186/s12957-023-02934-w

**Published:** 2023-03-01

**Authors:** Xiang Huang, Guoli Li, Jinjing Wang, Hong Zheng

**Affiliations:** Department of Pathology, The Affiliated Hospital of Zunyi Medical University, Zunyi City, Guizhou Province People’s Republic of China

**Keywords:** Inflammatory myofibroblastic tumour, Common bile duct, Clinical, Pathology

## Abstract

**Introduction:**

Inflammatory myofibroblastic tumour (IMT) of the common bile duct (CBD) is an extremely rare low-grade malignancy with various biological behaviours and a lack of specific clinical and histopathological features. Preoperative and intraoperative diagnosis are challenging, and a diagnostic delay may increase surgical complexity.

**Case presentation:**

We present the case of a 34-year-old male with no relevant medical history who presented with jaundice of 20 days of evolution. Histology and immunohistochemistry confirmed the diagnosis of an IMT with anaplastic lymphoma kinase (ALK)-1 expression. In addition, a review of the relevant literature revealed 13 published reports of biliary IMTs. The clinical history and histopathological features in these 13 cases were compared with those in our case to provide a comprehensive overview of the clinical manifestations and histopathological features of the disease.

**Conclusion:**

IMT of the CBD is an extremely rare low-grade malignancy that mainly occurs in middle-aged female patients. The main clinical manifestation is monosymptomatic jaundice. The reported tumours originated in the middle and lower segments of the CBD, with an average size of approximately 3.5 cm × 3.0 cm and tumour cells expressing smooth muscle actin (SMA), vimentin and ALK. Abnormal ALK expression and ALK gene rearrangement represent potential histopathological and differential diagnoses. A clear diagnosis by preoperative biopsy and intraoperative frozen section examination is critical and can significantly reduce surgical trauma. The prognosis is good, and very few patients experience recurrence or distant metastasis.

## Introduction

Inflammatory myofibroblastic tumours have been reported in various organs, such as the liver, intestine, spleen, kidneys, bladder, lungs, peritoneum and heart, and are most common in the lungs [1, 2]. However, an IMT in the common bile duct (CBD) is extremely rare. Only 13 reports of biliary IMTs have been published [3]. The disease lacks specific clinical manifestations and immune indicators, and approximately 50% of patients with IMTs have abnormal anaplastic lymphoma kinase (ALK) expression and ALK gene rearrangement [4]. The present study reports a case of IMT of the CBD positive for ALK rearrangement and reviews relevant literature to provide a comprehensive overview of the clinical manifestations and histopathological characteristics of the disease to deepen the understanding of the disease and improve preoperative and intraoperative diagnostic rates.

## Case presentation

**Fig. 1:**
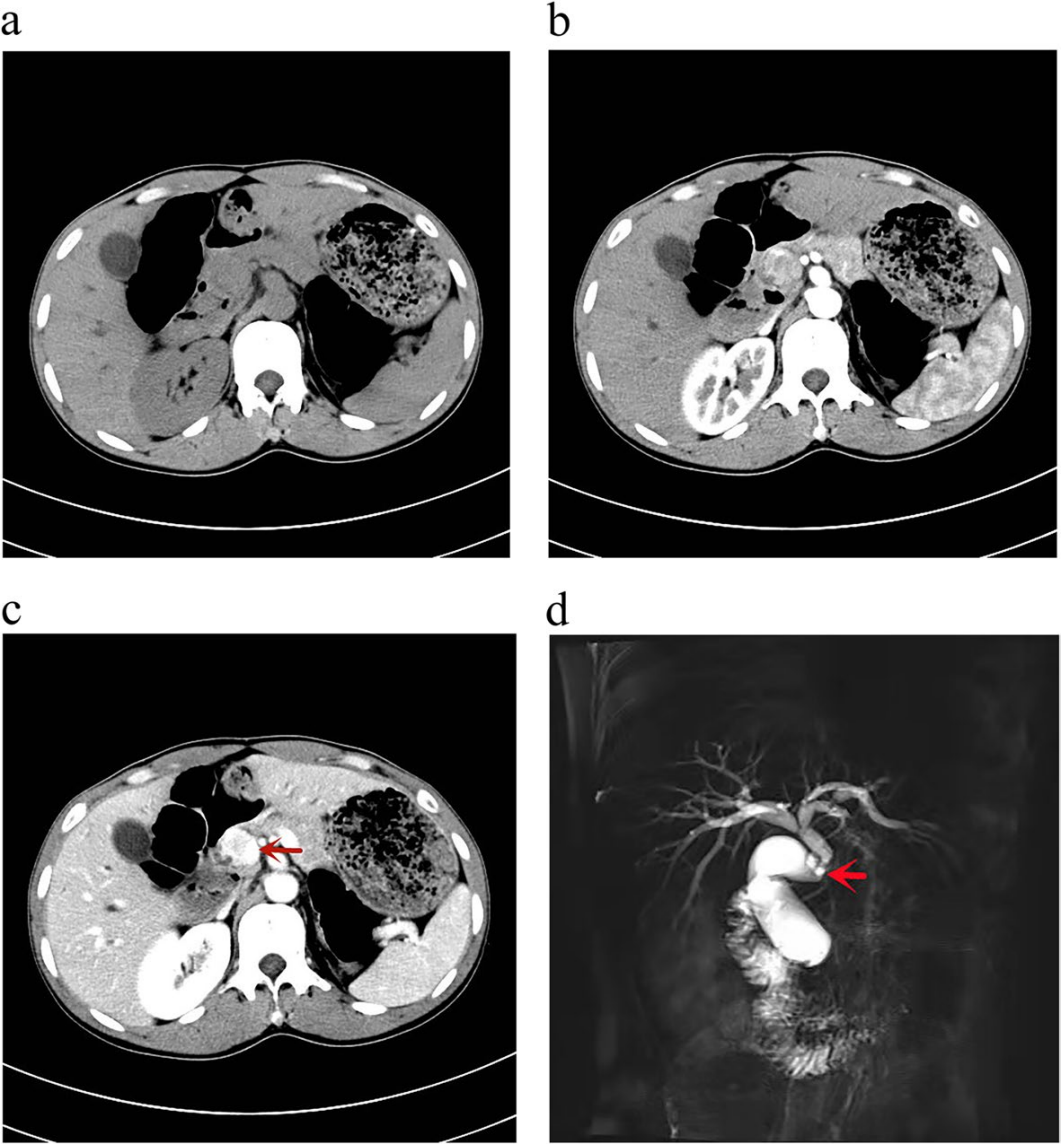
**a** Axial pre-contrast CT image. **b** axial post-contrast CT image in arterial phase. **c** Axial post-contrast CT image in portal venous image in delayed phase shows a heterogeneously enhancing mass at common bile duct (CBD) [red arrow in (**c**)]. **d** MRI image shows diffuse dilatation of the intrahepatic biliary radicles and proximal common bile duct (CBD) with narrowing noted at the middle of the CBD

We present the case of a 34-year-old male with no relevant medical history who presented with jaundice of 20 days of evolution. His examination revealed scleral and whole-body skin icterus. There was no abdominal tenderness, rebound tenderness, and the Murphy test was negative, with no palpable mass. There was no evidence of any other organomegaly or lymphadenopathy. His test results on admission were as follows: total bilirubin, 5.17mg/dl; direct bilirubin, 2.49 mg/dl; alkaline phosphatase, 152 U/L; and CA199, 58.5 U/ml. Viral serological tests for hepatitis A, B and C and HIV were negative. Computed tomography (CT) revealed the following: soft tissue nodules in the CBD with clear borders, inhomogeneous morphology, heterogeneous density, equal or slightly low-density changes and nodules bulging into the lumen of the CBD. In the venous phase, there was significant heterogeneous enhancement, which was more obvious than that in the arterial phase and showed gradual strengthening changes (Fig. 1a–c). Magnetic resonance cholangiopancreatography revealed a localized filling defect in the lower part of the CBD with luminal narrowing (Fig. 1d). The patient was evaluated by a multidisciplinary committee. The clinical diagnosis was obstructive jaundice with the possibility of bile duct cancer. Laparoscopic pancreaticoduodenectomy was performed. Intraoperatively, adhesions of the omentum and abdominal wall were observed, no ascites were observed in the abdominal cavity and severe cholestasis was observed in the liver. A mass approximately 1.5 × 1.5 cm in size was detected in the lower segment of the CBD. The gallbladder, duodenum, part of the stomach, CBD and head of the pancreas were removed. The bile duct and jejunum were anastomosed, the jejunum and pancreas were anastomosed and a silicone stent and drainage tube were placed. No biopsy was taken during the operation, and there was no biliary leakage.

**Fig. 2:**
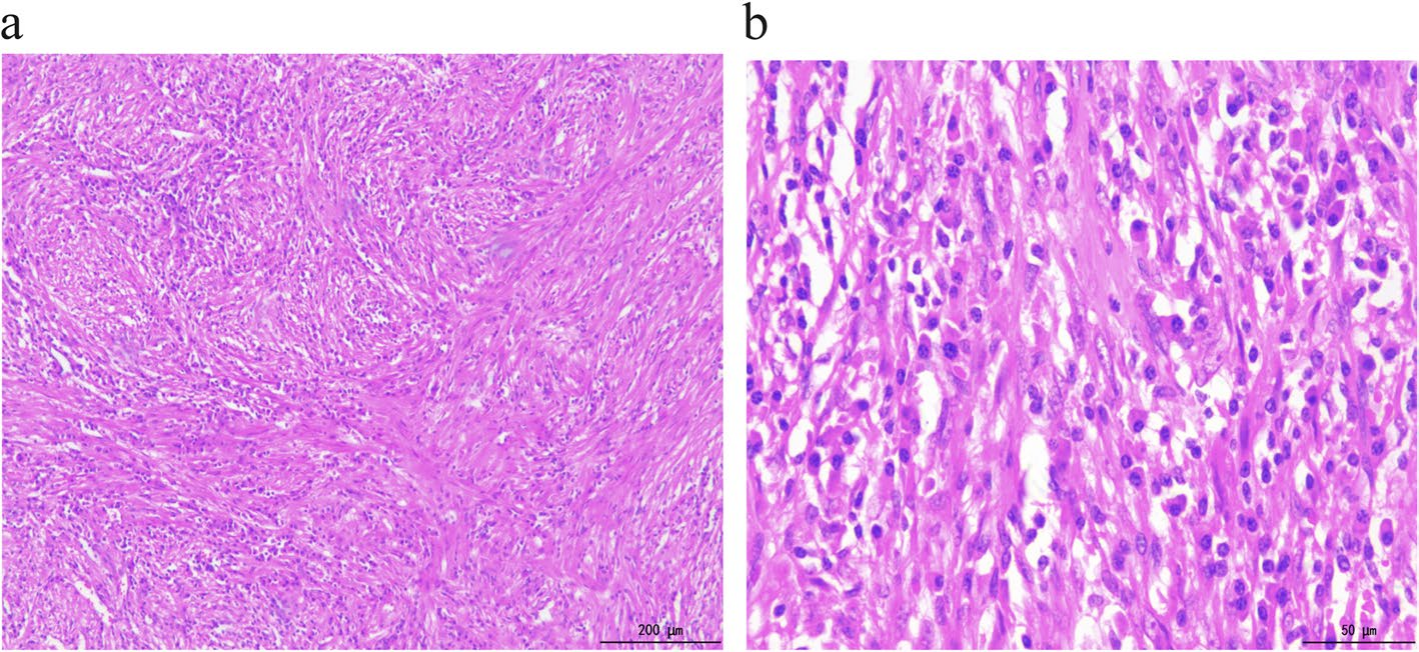
**a** Spindle-shaped cells arranged in fascicles in a background of chronic inflammatory cells (H&E 100×). **b** Spindle-shaped cells in a background of chronic inflammatory cells (H&E 400×)

**Fig. 3:**
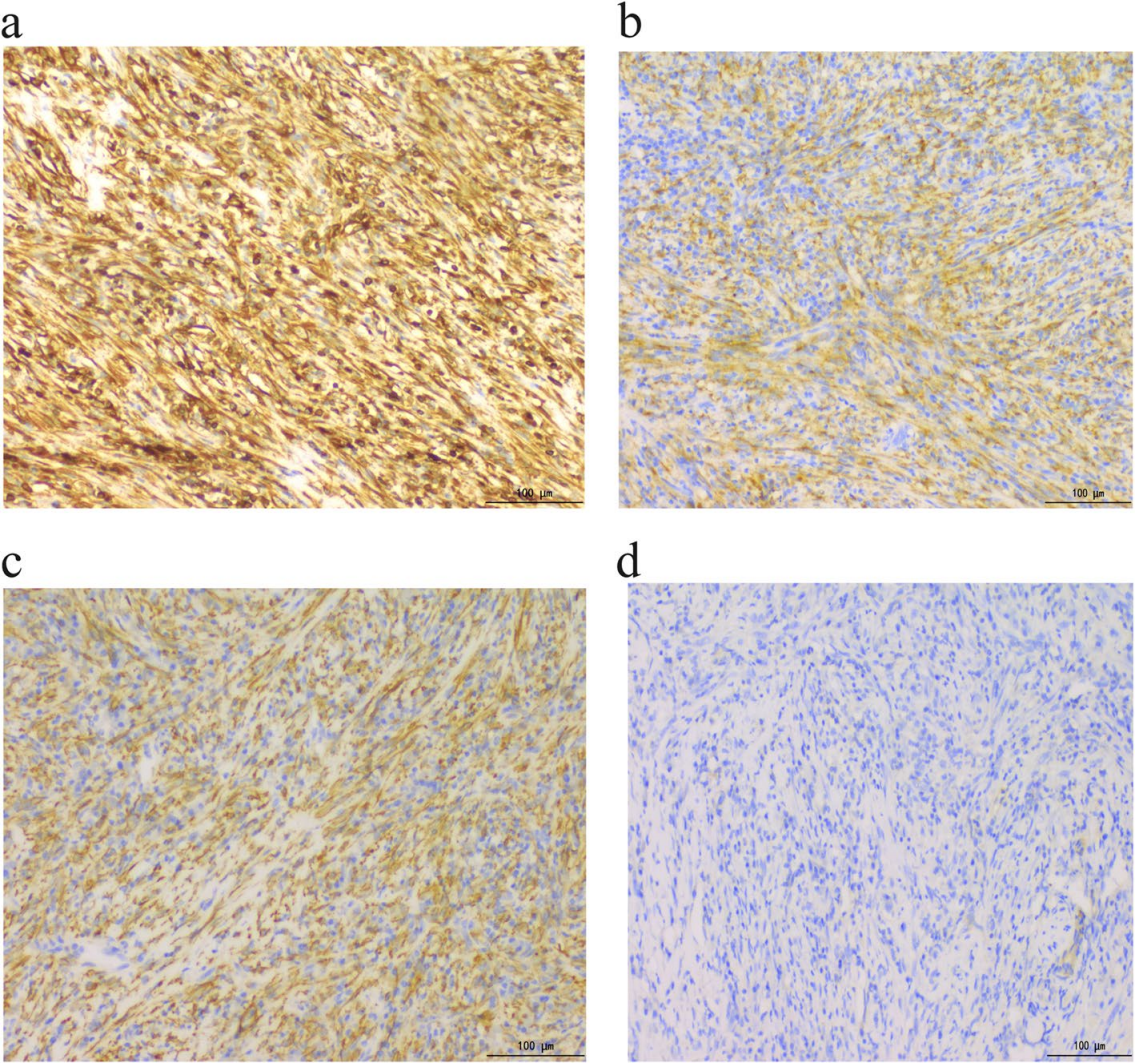
**a** Positive staining with vimentin (100×). **b** Positive staining with ALK (100×). **c** Positive staining with SMA (100×). **d** Negative staining with Dog-1 (100×)

**Fig. 4:**
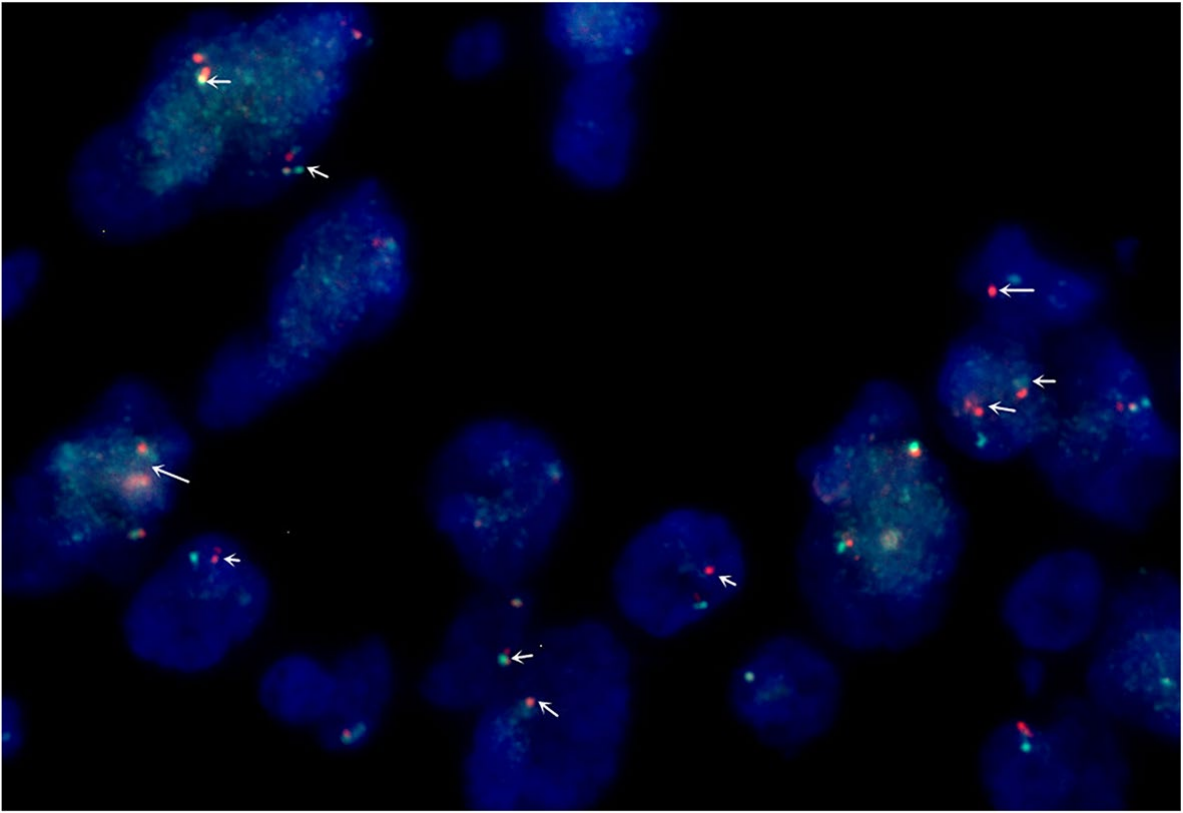
Immunofluorescence in situ hybridization (FISH) indicated that ALK was represented by a mainly orange-green signal or only an orange signal, indicating positive results

Pathological examination revealed a large 2 cm × 2 cm solid nodule at the CBD with a grey–yellow colour and an unclear surrounding border. Microscope showed that the myofibroblast spindle cells were tightly packed, with extensive infiltration of chronic inflammatory cells, slightly heterogeneous tumour cells and most regional nucleuses < 5/10 high-power fields (HPF) (Fig. 2a–b). No tumour involvement was observed in the duodenal ends, gastric segments, CBD ends or pancreatic margins. Furthermore, immunohistochemical analysis showed positivity for vimentin (Fig. 3a), ALK (Fig. 3b) and SMA (Fig. 3c). However, results for desmin, cytokeratin 7 (CK7), S-100, Dog-1 (Fig. 3d), cluster of differentiation 117 (CD117), cluster of differentiation 34 (CD34), succinate dehydrogenase (SDH) and cytokeratin (CK) expression were negative. The Ki-67 proliferative index was approximately < 20%, and the IgG4/IgG ratio was < 30%. Immunofluorescence in situ hybridization (FISH) indicated that ALK was represented by a mainly orange-green signal or only an orange signal, indicating positive results (Fig. 4). The postoperative pathological diagnosis was IMT. The patient was followed up for 3 years, and he is currently asymptomatic and without recurrence.

## Discussion

IMT was once regarded as a benign reactive tumour-like hyperplasia with spindle fibroblasts and myofibroblasts undergoing hyperplasia, accompanied by fibroblast infiltration as the main pathological feature [5]. Tumours of this pathologic morphology were first identified in the CBD by Haith et al. [6] in 1964 and first described in detail in the lungs by Bahadori et al. [7] in 1973; IMTs were subsequently reported in various organs [8, 9]. Due to histological particularities, these tumours have had many different names in the literature, such as inflammatory pseudotumours, plasma cell granulomas and plasma cell pseudotumours. With deeper understanding and research, the World Health Organization (WHO) (2002) eventually classified these soft tissue tumours as IMTs, namely, borderline tumours with occasional metastasis [10]. IMTs in the CBD are extremely rare, with only 13 previously reported cases in addition to our present case (Table 1). IMTs seem to be more common in middle-aged women; the male-to-female ratio is 2:5, and the age range of IMT patients is 6 to 71 years (average, 46.5 years). The main clinical manifestations are jaundice without other associated symptoms, but a few patients also show abdominal pain, emaciation and fever [11, 12, 21]. Biliary bleeding rarely occurs [17]. The patient in the present case was a 34-year-old male with jaundice as the predominant clinical manifestation, similar to the 13 previously reported cases. The levels of tumour markers are usually normal; however, our patient had a slightly elevated CA199 level. CA199 may be an effective tumour marker for the diagnosis of cholangiocarcinoma and monitoring the efficacy of treatment [22]. Therefore, cholangiocarcinoma should be considered as the primary differential diagnosis before surgery. Compared with the 13 previously reported cases, the reason for the elevated CA199 level in this case was unclear; however, due to the small number of reported IMT cases at present, the possibility of CA199 being related to IMT of the CBD cannot be excluded. Liver function examination revealed an elevated bilirubin level but normal or only slightly abnormal ALT and AST levels. ALT and AST levels were significantly abnormal in only one patient, reported by Fukushima et al. [13]. However, the bilirubin level was also elevated in the present case. In relation to the radiological findings of this entity, a common, narrow CBD is usually observed, but distinguishing it from cholangiocarcinoma is not possible; additionally, preoperative diagnosis is difficult. The patient in the present case also showed a CBD mass with stricture on imaging. Surgery remains the preferred treatment regimen. Interestingly, Lopez-Tomassetti et al. [18] reported local recurrence 4 years after choledochal IMT resection but chose corticosteroid treatment; complete tumour remission was reported 3 months after treatment, and 6 months after continued dose maintenance therapy, the patients remained well, with liver function markers in the normal range. This treatment approach represents a feasible secondary option for patients clinically unable to undergo surgery. Of the patient’s follow-up, the duration of follow-up ranged from 6 weeks to 19 years, and three have been reported to have experienced recurrence and distant metastases [12, 14, 18]. Our patient was followed up for 3 years, and he is currently asymptomatic and without recurrence. Walsh et al. [14] reported a 19-year follow-up of a patient who exhibited distant metastases occurring mainly in the lungs [12, 23], which is also the most common primary site of IMTs. Therefore, IMTs of the CBD are capable of recurrence and distant metastasis. Given the rarity of these tumours, long-term follow-up of patients with IMTs of the CBD is scarcely reported in the literature. Extrabiliary IMTs have the potential for local infiltration, recurrence and persistent local growth. Local recurrence may occur after many years; thus, strict follow-up after surgery is required [24]. In many cases, the behaviour of these tumours is complex and difficult to predict, especially when they occur at sites with few corresponding case reports in the literature. Although IMTs are known to be prone to local recurrence and may develop metastases, there are documented cases of both biliary and extrabiliary IMT patients without disease recurrence over prolonged follow-up periods. Arredondo Montero J. et al. [25] reported a case of a 2-month-old girl presenting with an ileal IMT. Long-term follow-up revealed that the patient remained asymptomatic and without recurrence after 15 years.

**Table 1:** IMTs in the CBD have been previously reported cases in addition to present case

Authors/publication year	Age/sex	Symptoms	Tumour marker	Liver function test	Location	Size (cm)	Immunohistochemistry	Treatment	Follow-up/outcome
E. E. Haith et al. [6] (1964)	6/M	Jaundice	─	─	Distal CBD	─	─	EHBD excision	5 months/NR
J. D. Stamatakis et al. [11] (1979)	13/F	Jaundice, abdominal pain		TB: 13.4 mg/dL	CBD, CD, CHD	3 cm × 3 cm	─	PD & celecoxib	21 months/NR
H. Ikeda et al. [12] (1990)	43/F	Fever, jaundice, significant weight loss	─	─	CHD, ProxCBD	─	─	Surgery	7 months/lung mets.
N. Fukushima et al. [13] (1997)	58/F	Abnormal liver function on physical examination	Normal	TB: 0.7 mg/dl, AST: 159 U/l, ALT: 454 U/l	Mid-lower CBD	2 cm × 2 cm	Vimentin (+), desmin (−), muscle-specific actin (−) and CD34 (+)	PD surgery	Not mentioned
S. V. Walsh et al. [14] (1998)	50/M	─	─	─	Proximal CBD	─	─	PD	19 years/metastasis
R. Sobesky et al. [15] (2003)	51/F	Jaundice	Normal	TB: 20.8mg/dL	Distal CBD	1.5 cm	CD34 (+), desmin (+), S-100 (+), SMA+	PD	2 years/NR
Antonio Martín Malagón et al. [16] (2006)	51/F	Jaundice	Normal	TB/DB: 11.3/9.3 mg/dl	Distal CBD	0.7 cm × 0.7 cm	SMA (+), S100 (−), vimentin (+), CD45 (+), CD68 (+)	PD	1 year/NR
Anuradha Sekaran et al. [17] (2006)	17/F	Jaundice, abdominal pain, black stool	─	TB/DB: 3.9/2.3 mg/dL	Distal CBD	─	SMA (+), S100 (+), vimentin (+)	PD	6 weeks/NR
E M López-Tomassetti Fernández et al. [18] (2006)	55/F	Jaundice	─	─	Distal CBD	5 cm × 5 cm	─	Cortical steroid therapy	3 months/tumor regression
Bassam Abu-Wasel et al. [19] (2012)	55/M	Jaundice	Normal	TB: 20.8mg/dL	Distal CBD	3 cm × 3 cm	─	PD	14 months/NR
K. Vasiliadis et al. [20] (2013)	70/F	Jaundice	Normal	DB: 14.9 mg/dl	Mid-distal CBD	3 cm × 3 cm	─	PD	8 months/NR
Aureen D’Cunha et al. [21] (2016)	12/F	Jaundice, abdominal pain	─	TB/DB: 13.86 /12.1 mg/dL	Distal CBD	4 cm × 4 cm	SMA (+), ALK (+)	PD	9 months/NR
Ritu Verma et al. [3] (2019)	24/F	Jaundice	Normal	TB/DB: 5.8/2.9 mg/dL	Mid CBD	10 cm × 6 cm	Desmin (+), SMA+, (CK), CD34 (−), CD117 (−), DOG1 (−), ALK1 (−)	PD	Not mentioned
Present case	34/M	Jaundice	CA199: 58.5 U/ml	TB/DB: 5.17/2.49 mg/dL	Mid CBD	2 cm × 2 cm	Vimentin (+), ALK (+), SMA (+), desmin (−), CK7 (−), S-100 (−), CD34 (−), DOG1 (−), CD117 (−), Ki-67 (20%+)	PD	3 years/NR

*M* male, *F* female, *CBD* common bile duct, *PD* pancreatoduodenectomy, *CD* cystic duct, *CHD* common hepatic duct, *EHBD* extrahepatic bile duct, *TB* total bilirubin, *DB* direct bilirubin, *NR* no recurrence

Analysis of tumour pathological characteristics revealed the following: the reported tumours originated in the middle and lower segments of the CBD with an average size of approximately 3.5 cm × 3.0 cm, and the largest tumour, reported by Verma et al., was approximately 10 cm × 6 cm in size [3]. Gross lesions are greyish-white, greyish-yellow, firm and nodular. A small number of the lesions are mucoid [13]. On histomorphology, three types of IMT growth patterns are observed: (1) the mucus-type pattern, (2) the cellular compact spindle cell pattern, and (3) the fibre-type pattern. The dense spindle cell pattern is the most common, and the different histopathological morphologies are not associated with prognosis, which is consistent with the findings reported by Coffifi et al. [8]. The tumour cells express SMA and vimentin; partially express desmin, CD34, CD68 and ALK; and do not express CK or S100. Abnormal ALK expression and ALK gene rearrangement represent histopathological and differential diagnosis, and ALK rearrangement is seen in approximately 50% of patients [2]. ALK is a tyrosine kinase receptor, and ALK expression plays an important role in gene rearrangement in the development of IMT [26]. In the present case, the tumour occurred in the middle part of the CBD, and the tumour cell growth pattern was mainly of the dense spindle cell type. The immunohistochemical expression pattern in this case is consistent with that in the previously reported case with ALK rearrangement.

Differential diagnosis of IMT of the CBD includes cholangiocarcinoma, gastrointestinal stromal tumour (GIST), leiomyoma and fibromatosis [3]. (1) Extrahepatic cholangiocarcinoma occurs predominantly in elderly individuals and often presents with systemic manifestations of malignancy. Immunohistochemistry can assist in identification. (2) GIST usually involves the ampullary/periampullary region. These cells are typically positive for vimentin and SMA but negative for CD117 and DOG1. (3) The boundaries of leiomyomas are clear, and these tumour cells present a rich eosinophilic fibrous cytoplasm and smooth muscle tumour cells that are regularly arranged, woven and have no obvious inflammatory cell infiltration. Immunohistochemistry can assist in identification. (4) IgG4-related sclerosing inflammatory lesions are reported in most cases of IMT of the distal CBD and show a strong association with sclerosing lymphoplasmacytic pancreatitis or autoimmune pancreatitis. In our case, interstitial plasma cells had an IgG4/IgG < 30%. (5) In fibromatosis, more myofibroblasts are seen in the lesions, but the cells are mainly arranged in parallel broad bundles with slightly wavy curves. Obvious infiltration of the surrounding skeletal muscle, tendon and tendon membrane is observed. Inflammatory cell infiltration is not as diffuse as that in IMT; fibromatosis is also associated with CD34 positivity and catenin negativity and no expression of SMA or MSA.

All patients were subsequently diagnosed with infiltrating biliary or peribiliary pseudotumours on laparotomy. No patient was, in fact, thought to have a nonneoplastic lesion before biliary diversion. Extrahepatic biliary resection was performed in 14 patients. However, the final pathological diagnosis was IMT. IMTs of CBD are rare lesions that are often mistaken for aggressive malignancies, and although their appearance is nonspecific, a diagnosis may be made by imaging-directed core biopsy. If biopsy is performed preoperatively or intraoperatively, the trauma of surgery can be reduced.

## Conclusion

Compared to GIST, leiomyoma and fibromatosis, IMT is considered a less malignant mesenchymal tumour occurring predominantly in children or adolescents or, in the case of IMT of the CBD, in middle-aged female patients. IMT of the CBD is characterized by monosymptomatic jaundice and a tumour in the middle or lower segment of the CBD, with an average size of approximately 3.5 cm × 3.0 cm and diverse histological features. The notable immunohistochemical characteristics of these tumours are SMA expression and partial expression of desmin, vimentin and ALK. Surgical excision is currently considered the treatment of choice for IMT. However, there is some evidence of the effectiveness of corticosteroid therapy. Although IMT of the CBD might be considered in the differential diagnosis of an obstructing extrahepatic biliary lesion, there are no distinctive clinical or imaging features that allow it to be differentiated from cholangiocarcinoma before laparotomy. Awareness of this rare but benign entity, however, might prompt a more limited operative approach when intraoperative resection margins of palpable disease are repeatedly negative for tumour cells. A clear diagnosis by preoperative biopsy and intraoperative frozen section examination is critical and can significantly reduce surgical trauma. The prognosis is good, and very few patients experience recurrence or distant metastasis.

## Data Availability

All the data regarding the findings are available within the manuscript.

## Abbreviations

CT: Computed tomography
MRI: Magnetic resonance imaging
CBD: Common bile duct
M: Male
F: Female
PD: Pancreatoduodenectomy
NR: No recurrence
CD: Cystic duct
CHD: Common hepatic duct
EHBD: Extrahepatic bile duct
ALK: Anaplastic lymphoma kinase
SMA: Smooth muscle actin
CK: Cytokeratin
CK7: Cytokeratin 7
SDH: Succinate dehydrogenase
CD34: Cluster of differentiation 34
CD117: Cluster of differentiation 117
